# Pituitary Adenylate Cyclase-Activating Polypeptide 6-38 Blocks Cocaine- and Amphetamine-Regulated Transcript Peptide-Induced Hypophagia in Rats

**DOI:** 10.1371/journal.pone.0072347

**Published:** 2013-08-15

**Authors:** Jonathan R. Burgos, Britt-Marie Iresjö, Ulrika Smedh

**Affiliations:** Department of Surgery, Institute of Clinical Sciences, Sahlgrenska Academy, Sahlgrenska University Hospital, Gothenburg, Sweden; University of Rouen, France

## Abstract

Cocaine- and amphetamine-regulated transcript peptides (CARTp) suppress nutritional intake after administration into the fourth intracerebral ventricle. Recent *in vitro* studies have shown that PACAP 6-38, a pituitary adenylate cyclase-activating polypeptide (PACAP) fragment, could act as a competitive antagonist against CARTp 55-102 on a common CARTp-sensitive receptor structure. Here, we show for the first time *in vivo* that the reduction in solid food intake induced by exogenous CARTp 55-102 (0.3 nmol: 1.5 µg) administered fourth i.c.v. is blocked by pretreatment with PACAP 6-38 (3 nmol). The PACAP 6-38 fragment had no effect by itself either when given into the fourth ventricle or subcutaneously. Although effective to block the CARTp-effect on feeding and short-term body weight, PACAP 6-38 failed to attenuate CARTp-associated gross motor behavioral changes suggesting at least two CARTp-sensitive receptor subtypes. In conclusion, PACAP 6-38 acts as a functional CARTp antagonist *in vivo* and blocks its effects on feeding and short term weight gain.

## Introduction

Cocaine-and amphetamine-regulated transcript and its peptides (CARTp) are widely distributed in many areas of the brain, and present in areas that are involved in reward, stress, controls for feeding and gastrointestinal physiology [1]. CARTp subsequently induces a variety of physiological and behavioral effects upon central administration including inhibition of food intake [2], [3], gastrointestinal function [4], [5], stress response [6] and neuroendocrine controls [7], [8]. The specific identity and neuroanatomical location for CARTp receptor(-s) are not fully known. *In vitro* studies on cultured neurons show that CARTp inhibits voltage-dependent Ca^2+^ signaling [9] which is blocked by pertussis toxin, indicating a G_i/o_ protein-coupled event [9], [10]. Jones and Kuhar showed that CARTp binding affinity was reduced by the addition of a GTP analogue but not of an ATP analogue, further suggesting the involvement of a G-protein coupled receptor as a putative target site [11]. Intracerebroventricular (i.c.v.) injection of CARTp increased CREB phosphorylation in CRF-containing neurons of the paraventricular hypothalamic nucleus (PVN) [7] and induced phosphorylation of NMDA receptors by protein kinase A and protein kinase C pathways. Together, evidence suggests that CARTp acts on a G_i/o_ -protein-coupled receptor to produce physiological effects via protein kinases A and C as second messengers, although the receptor has not yet been cloned.

In lack of a receptor protein sequence, attempts to identify anatomical binding sites on sectioned tissues by autoradiography using labeled CART peptides as ligands have failed so far due to technical problems with high unspecific background [12], [13]. In one study of mouse hypothalami using a GFP-fusion technique, staining was seen in the periventricular area suggesting binding sites for CARTp in this location [13]. Some further indication as to where the relevant target sites for CARTp effects on food intake and gastrointestinal functions may be situated is provided by functional studies. Lateral as well as fourth i.c.v. application of CARTp in a dose range of 1–2 µg inhibits food intake as shown in a number of studies [2]–[5], [12], [14]–[19]. Aja et al. [2] designed a study to identify the location of the CARTp-responsive substrate(-s) on feeding. By placing a grease plug in the aqueduct and delivering CARTp to both the lateral and fourth ventricles, they showed that the food intake inhibitory effect is elicited from the dorsal hindbrain rather than from a forebrain substrate target.

Recently, PACAP 6-38–a PACAP (pituitary adenylate cyclase-activating polypeptide) non-stimulating competitive antagonist–was shown to exhibit low-affinity binding to a receptor structure in cultured PC12 cells, and binding was displaced competitively by the physiologically active CART peptide fragments CARTp 55-102 as well as CARTp 61-102. In addition, PACAP 6-38 blocked the CARTp-induced phosphorylation of ERK in differentiated cells [20]. These findings indicate that PACAP 6-38 acts as an *in vitro* competitive antagonist to CARTp fragments. The discovery of a new receptor ligand candidate is of great value, not only since it may be used to further the anatomical determination of putative CARTp binding sites, but its antagonistic properties may also be of value to firmly establish the possible role of *endogenous* CARTp. The discovery of a ligand that acts competitively to CARTp on a common receptor structure, albeit with lower affinity than the agonist, may help to facilitate structural identification of the putative CARTp receptor protein and for other, high-affinity ligands, to be developed.

It cannot be generally assumed that a ligand that has antagonistic properties on cultured cells *in vitro* in an ideally set experimental microenvironment retains such properties on a range of physiological or behavioral events under *in vivo* conditions in the intact animal [21]. Since the inhibition of ingestive behavior is considered to be an important functional feature of central CARTp, the aim of the present study is to specifically test whether PACAP 6-38 serves as a functional CARTp 55-102 antagonist on solid food intake in the rat. The fourth ventricle will be used as a route for drug delivery based on previous reports highlighting the hindbrain as a key location for CARTp inhibition of nutrient intake [2], [5], [18]. The endogenous fragment CARTp 55-102 is used, since its effect to inhibit food intake after fourth i.c.v. injection is well established [2], [5], [14], [16], [22]–[24] and it is competitively displaced by PACAP 6-38 *in vitro* [20]. We show for the first time that PACAP 6-38 acts as a functional CARTp antagonist *in vivo* and blocks CARTp-induced hypophagia and short term weight loss.

## Materials and Methods

### Ethics Statement

The study protocols were approved by the Gothenburg Animal Ethics Committee and in accordance with national and EU regulations for animal welfare.

### Animals

Male Sprague-Dawley rats (Scanbur AB, Sollentuna, Sweden) were housed singly in plastic cages under conditions of controlled temperature (20±1°C) and relative humidity (50±10%) on a reversed 12∶12 h light cycle (lights off at 10 AM). The animals were weighed daily at 8 AM throughout the study and were provided with tap water and standard chow (R34; Lantmännen, Stockholm, Sweden) *ad libitum* unless else specified. On testing days, rats were moved to similar cages equipped with wire floors to which they had been previously habituated.

### Surgery

Rats weighing an average of 340 g (range 312–371 g) were anesthetized with a mixture of xylazine (8.6 mg/kg) and ketamine (57 mg/kg), injected 1 ml/kg body weight i.m. in the hind leg prior to stereotaxic surgery. The surgeries were performed under aseptic conditions. The animal was placed in a stereotaxic frame (Kopf Instruments, Tejunga, CA), and a local anesthetic (mepivacaine; Carbocain adrenalin, 10 mg/ml +5 µg/ml; AstraZeneca AB, Södertälje, Sweden) was injected in the skin and subcutaneous tissue of the scalp. After this, the skull was exposed, a small hole was created in the skull bone using a 1.8 mm trephine and a chronic guide cannula (10.0 mm×21 G; in-house made) aimed at the fourth ventricle was implanted and attached by means of anchor screws and dental acrylic, as previously described [25]. The animals were weighed and handled daily for one week following cannula placement but did not undergo any other procedures.

Correct cannula placements were verified by a functional test performed five days prior to the first experimental testing session. The animals were injected with 210 µg 5-thio-D-glucose (5-TG; Carbosynth Limited, Berkshire, UK) dissolved in sterile water. Blood glucose measurements were obtained with a standard glucometer (FreeStyle Precision; Abbott Laboratories AB, Solna, Sweden) just before and one hour after 5-TG injection. A doubling in blood glucose concentration compared to baseline was taken as evidence of a correct placement [26]. Animals that did not show the required blood glucose response to 5-TG were not included in the i.c.v. experiments.

### Drugs

Synthetic CART 55-102 peptide (rat) and PACAP fragment 6-38 (human, ovine, rat) were both obtained from American Peptide Company (Sunnyvale, CA). The peptides were dissolved in sterile 0.9% saline, divided into small aliquots and frozen (−20°C). A fresh aliquot was defrosted on each experimental day and any excess was discarded. Sterile saline was used as the vehicle in all experiments (B. Braun Melsungen AG; Melsungen, Germany).

### Experimental Design and Procedures

For experiments 1 and 2, the animals were gently restrained by hand, and a 27G injection needle was inserted via the guide and into the fourth ventricle. The injection needle was attached to a 10 µl Hamilton syringe via a 20 PE tube, and injections were administered into the fourth ventricle over 30 s. The needle was left in place for another 30 s to avoid any risk of back flush, after which it was removed and replaced with an obturator. In experiment 3, injections of drug or vehicle were given subcutaneously (s.c.) in the skin of the back.

The animals (n = 8 in experiments 1 and 2, n = 7 in experiment 3) served as their own control subjects. Each animal received each combination of drugs, or vehicle, once and in random order. The experiments were performed every third day to allow for any carry-over effects of the drugs to be washed out.

In experiment 1, we investigated whether PACAP 6-38 acts to block dorsal hindbrain CARTp 55-102-induced inhibition of food intake in the rat. Eight rats with confirmed cannula placements were injected fourth i.c.v. on each testing day 20 min prior to lights out and food access: 1.5 µl of vehicle or PACAP 6-38, followed ten minutes later with 1.5 µl of CARTp 55-102 (0.3 nmol: 1.5 µg) or vehicle. The following PACAP 6-38 doses were used: 0.3 nmol, 0.6 nmol, 3 nmol. Food consumption was measured at 2, 5 and 22 h.

In experiment 2, possible effects by PACAP 6-38 by itself to change food intake were investigated. Injections of 3 µl drug or vehicle were administered fourth i.c.v. 20 min prior to lights out and food access. The following doses were given: 0.3 nmol, 0.6 nmol, or 3 nmol PACAP 6-38. Food intake was measured at 2, 5 and 22 h.

A potential peripheral feeding effect of PACAP 6-38 has, to our knowledge, not yet been fully elucidated. In experiment 3 our aim was to test whether the PACAP 6-38 antagonistic effect in experiment 1 was due to a central mechanism (i.e. that the observed phenomenon in experiment 1 was not caused by PACAP 6-38 crossing the brain-blood barrier and exerting a confounding counter-effect from a peripheral primary target). Therefore, PACAP 6-38 was given s.c. in a dose range corresponding to that administered in experiments 1 and 2. To further ensure that the highest centrally administered dose was not just simply sub-threshold for a peripheral action, the dose range was extended to include an additional PACAP 6-38 treatment of 6 nmol (twenty times the effective fourth i.c.v. CARTp and twice the highest PACAP 6-38 doses). Twenty minutes prior to lights off and food access, 0.4 ml drug or vehicle was injected. Food intakes were recorded at 2 h and 5 h after injection.

### Behavioral Measurements

On each testing day, the animals had free access to water throughout the experiment, and free access to food until 1 h prior to lights off when the food hoppers were emptied. The animals were placed in individual testing cages lined with pre-weighed aluminum foil and fitted with wire mesh floors to allow for the collection of food spillage. A pre-weighed portion of standard chow was placed in the feeder of the respective cage just prior to lights off. At defined time points after lights out/food access (2 h, 5 h, and 22 h for experiments 1 and 2; 2 h and 5 h for experiment 3) the food was quickly removed from the food hoppers and immediately replaced with another pre-weighed portion. Each food exchange procedure never exceeded five minutes for the entire group and was performed quietly under minimal additional light (a single 25 Watt red incandescent darkroom bulb) in order to minimize disturbances to the animals. The solid food intakes as well as the dried spillages were determined gravimetrically (Precisa 1600C; Precisa Gravimetrics AG, Dietikon, Switzerland), and corrected food consumption for each time point was calculated. Additionally, in experiments 1 and 2, the presence of typical motor behavior (previously described by Aja et al. [2], [14], [15], Kristensen et al. [12] and Kimmel et al. [27]) in response to CARTp and/or PACAP 6-38 was observed. It was noted whether motor effects were present or not just before lights off/food access and in connection with the food changing procedure at 2 h, 5 h, and 22 h.

### Statistical Evaluation

Solid food intake at each time point was evaluated with repeated measures ANOVA. Tukey’s post-hoc tests for multiple comparisons were performed where applicable. *P*-values less than 0.05 were regarded as significant.

## Results

### Experiment 1

**Figure 1:**
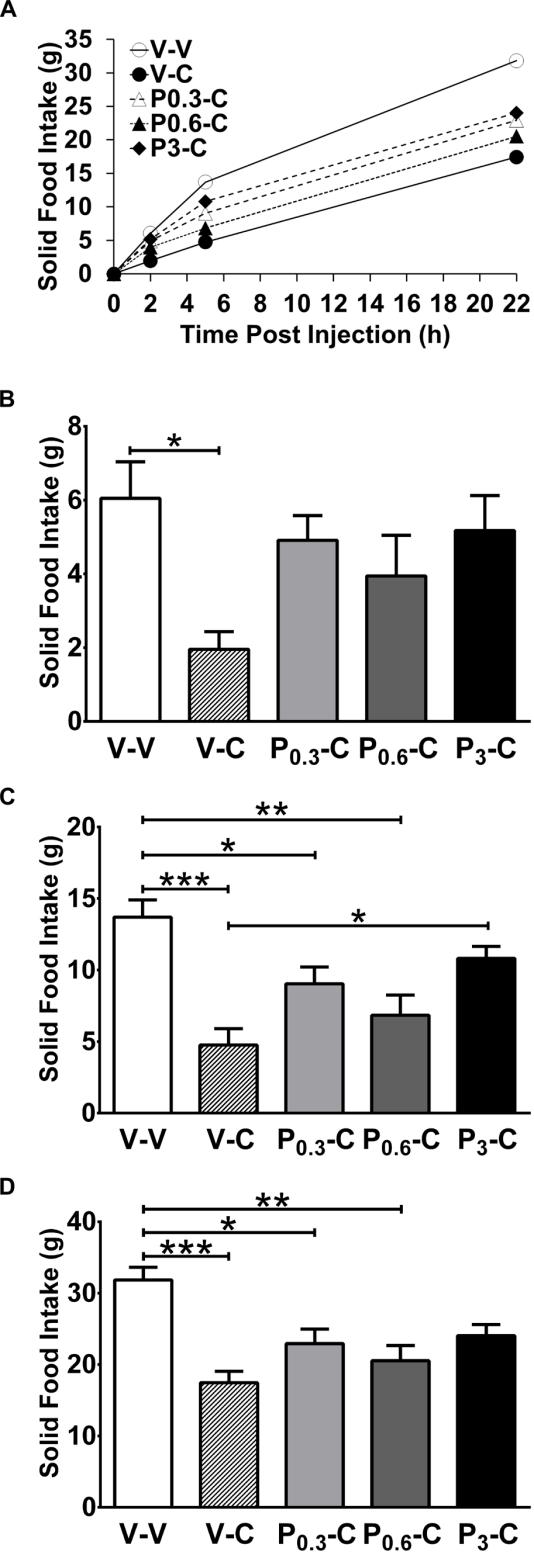
Fourth i.c.v. PACAP 6-38 blocks CARTp-induced reductions in solid food intake. Male Sprague-Dawley rats (n = 8) were pretreated with varying doses of PACAP fragment 6-38 (range 0.3–3 nmol) or vehicle prior to dosing with 0.3 nmol CARTp or vehicle. (A) Solid food intake measures (mean g ±SE) from the entire 22 h observation period. Cumulative solid food intake was recorded at 2 h (B), 5 h (C), and 22 h (D) following peptide injections and food presentation. CARTp significantly inhibited food intake 2–22 h after injection (Fig. 1A–D). Pretreatment with 3 nmol PACAP 6-38 completely blocked the effect after 5 h (Fig. 1 C). Significances from Tukey’s post hoc test after repeated measures ANOVA are indicated above: *p<0.05; **p<0.01; ***p<0.001. The treatment abbreviations are V = saline vehicle; C = 0.3 nmol CARTp; P_0.3_ = 0.3 nmol PACAP 6-38; P_0.6_ = 0.6 nmol PACAP 6-38; P_3_ = 3 nmol PACAP 6-38.

Repeated measures ANOVA revealed an overall effect of treatment on solid food intake for each of the time points: (t = 2 h: F_(4,28)_ = 3.32, p<0.02; t = 5 h: F_(4,28)_ = 9.47, p<0.0001; t = 22 h: F_(4,28)_ = 7.34, p<0.0005). Animals receiving CARTp consumed about half as much food on average compared to the control group (vehicle/CARTp vs. vehicle/vehicle group; Fig. 1). Post hoc Tukey’s test showed that at all the three time points, there was a significant suppression of food intake in response to CARTp (vehicle/CARTp vs. vehicle/vehicle groups: p<0.05 at 2 h and p’s<0.001 at 5 h and 22 h, respectively, Fig. 1B–D). The lower PACAP 6-38 doses (0.3 and 0.6 nmol) were effective at attenuating CARTp-induced feeding suppression for up to 5 hours (Fig. 1B and C). After the highest (3 nmol) PACAP 6-38 dose, the PACAP 6-38 -induced attenuation of the CARTp effect was sustained throughout the 22 h observation period (Fig. 1D, 3 nmol PACAP 6-38/CARTp vs. vehicle/vehicle, p>0.05). Moreover, at the 5 h time point, pretreatment with 3 nmol PACAP 6-38 completely blocked the effect of CARTp (3 nmol PACAP 6-38/CARTp vs. vehicle/vehicle, p>0.05 and 3 nmol PACAP 6-38/CARTp vs. vehicle/CARTp, p<0.05, Fig. 1C). These results notwithstanding, there was no statistical difference with regard to dose between the three PACAP6-38/CARTp conditions (0.3, 0.6 or 3 nmol) at any time point.

**Figure 2:**
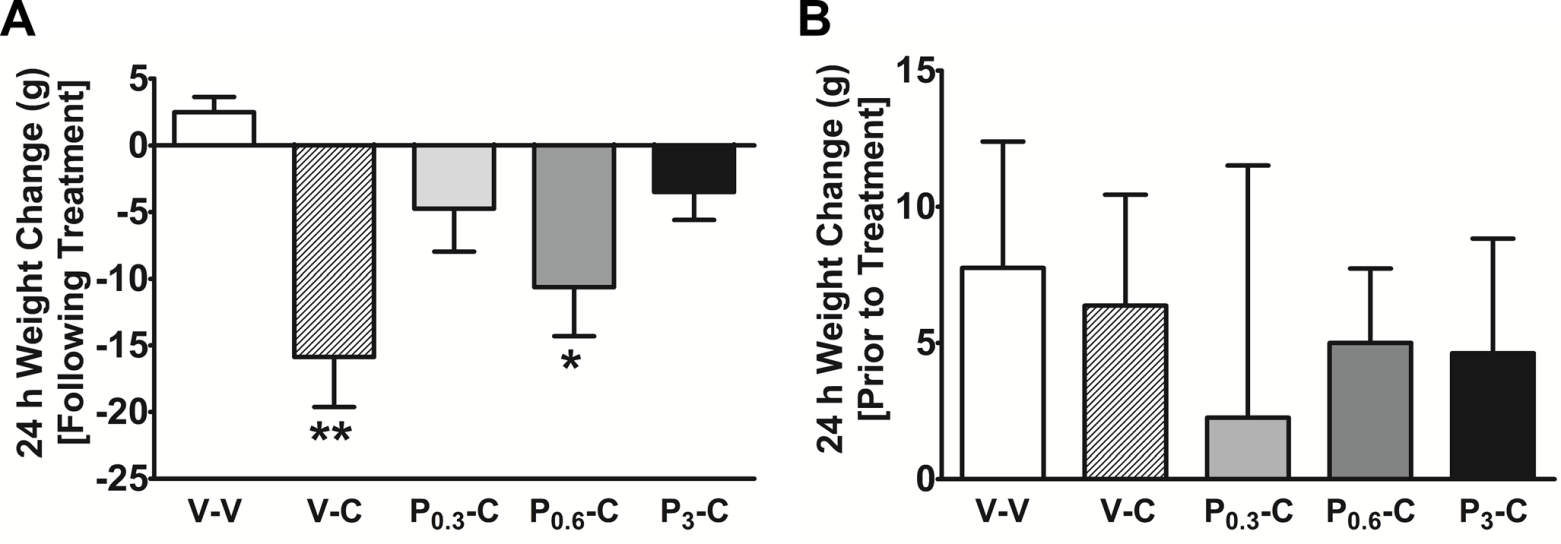
PACAP 6-38 blocks CARTp-induced reduction in body weight. Effects by fourth i.c.v. drug treatment on body weight changes (mean g ± SE). (A) The animals receiving CARTp had significantly decreased body weight on the day after fourth i.c.v. injections (22 h post injection); however, PACAP 6-38 (P) was able to mitigate the weight loss effects observed in the animals. (B) During the 24 h preceding the next treatment session (i.e., 2 days later), all animals again displayed stable positive weight changes that were similar across groups, regardless of the preceding treatments. Significance from Tukey’s post-tests after repeated measures ANOVA are indicated: *p<0.05; **p<0.01; ***p<0.001. All significant differences shown in the figure are vs. the vehicle control condition.

CARTp treatments were also observed to result in a significant decrease in overnight body weight; the body weight data are summarized in Fig. 2. Repeated measures ANOVA showed an overall effect of treatment on over-night body weight (t = 22 h, F_(4,28)_ = 5.34, p<0.005). Post hoc Tukey’s test showed that in the vehicle/CARTp treated group, the over-night body weight was significantly lowered (p<0.01) vs. the vehicle/vehicle treated animals. Similar to the food consumption findings, PACAP 6-38 pretreatments lessened the magnitude of the CARTp-induced change in body weight. In fact, post hoc comparisons using Tukey’s test showed that the 3 nmol PACAP 6-38 dose antagonized the CARTp inhibitory effect on body weight completely (vehicle/CARTp vs. vehicle/vehicle, p<0.05; vehicle/CARTp vs. 3 nmol PACAP 6-38/CARTp, p<0.05; and 3 nmol PACAP 6-38/CARTp vs. vehicle/vehicle, p>0.05). Continued monitoring of body weight showed that the differences in body weight were short-lived and limited to only 24 h after treatment. On the day prior to the next injection session, differences in body weight change were no longer present (F_(4,28)_ = 1.36, p = 0.29, ns). These latter observations were taken as confirmation that the interval between testing sessions was sufficient.

All animals treated with fourth i.c.v. CARTp regardless of whether PACAP 6-38 pretreatment was given or not exhibited motor effects shortly after injection - wobbly gait and prone resting position. Thus, PACAP 6-38 pretreatments were ineffective in blocking CARTp effects on motor behavior.

### Experiment 2

The same PACAP 6-38 doses as in experiment 1 were administered fourth i.c.v. in the absence of CARTp to rule out any possible intrinsic central effects the peptide may have had on food intake. The food intake data are summarized in Table 1. Repeated measures ANOVA showed no overall effects of fourth i.c.v. PACAP 6-38 treatment on solid food intake after 2 h (F_(3,21)_ = 0.14, p = 0.93), 5 h (F_(3,21)_ = 1.02, p = 0.40) or 22 h (F_(3,21)_ = 2.21, p = 0.12, ns), confirming that PACAP 6-38 had no effects by itself on food intake upon central administration in the current dose range.

**Table 1:** Fourth i.c.v. administration of PACAP 6-38 does not by itself affect solid food intake.

	Solid Food Intake (g)		
Treatment	2 hours	5 hours	22 hours
Vehicle	6.42±0.69	13.0±0.61	29.7±1.30
PACAP 6-38 0.3 nmol	6.56±1.13	11.4±0.86	26.3±1.93
PACAP 6-38 0.6 nmol	6.25±0.79	11.9±0.64	27.6±0.91
PACAP 6-38 3 nmol	6.87±0.57	11.6±0.64	25.6±0.92

PACAP 6-38 (0.3–3 nmol) did not affect food intake by itself vs. vehicle after fourth i.c.v. administration in Experiment 2. Cumulative solid food intake in rats (n = 8) expressed as mean (g ±SE).

In addition, no changes in motor behavior were observed in any of the animals at any dose or time point in response to fourth i.c.v. injection of PACAP 6-38.

### Experiment 3

Solid food consumption measures after s.c. injections of PACAP 6-38 are shown in Table 2. There was an overall effect of treatment on solid food intake after 2 h (F_(4,24)_ = 3.10, p<0.05) but not after 5 h (F_(4,24)_ = 2.23, p>0.05). However, Tukey’s post-hoc test showed no significant differences between any of the PACAP 6-38 doses and the vehicle condition, at any time point, indicating that PACAP 6-38 had no effects by itself on food intake after peripheral application in the extended fourth i.c.v. dose range. It was additionally noted that s.c. administered PACAP 6-38 did not induce changes in motor behavior.

**Table 2:** Peripheral administration of PACAP 6-38 does not affect solid food intake.

	*Solid Food Intake (g)*	
Treatment	*2 hours*	*5 hours*
Vehicle	5.14±0.71	13.4±1.01
PACAP 6-38 0.3 nmol	5.91±0.42	13.7±1.12
PACAP 6-38 0.6 nmol	4.75±0.45	12.7±0.55
PACAP 6-38 3 nmol	5.11±0.88	12.7±1.18
PACAP 6-38 6 nmol	8.07±1.65	15.5±1.59

PACAP 6-38 (0.3–6 nmol) did not affect solid food intake after s.c. administration as compared to vehicle in Experiment 3. Cumulative solid food intake in rats (n = 7) expressed as mean (g) ±SE.

## Discussion

In the present study we show for the first time that PACAP 6-38 acts as a functional *in vivo* antagonist effectively blocking CARTp 55-102 -induced hypophagia after fourth i.c.v. administration. There was a robust CARTp effect that was sustained for 22 h, resulting in suppression of solid food intake by approximately 50% versus controls. PACAP 6-38 pretreatments, at the lower doses (0.3 and 0.6 nmol), were able to mitigate CARTp feeding effects for 2 h while the high dose (3 nmol PACAP 6-38) complete blockade persisted for between 5 and 22 h (Fig. 1 C and D). We replicated effects showing a short-lasting reduction in weight gain [23] in response to fourth i.c.v. CARTp that was blocked by 3 nmol PACAP 6-38 pretreatment (Fig. 2A).

The observation that PACAP 6-38 blocks effects of exogenously delivered CARTp *in vivo* (Fig. 1) parallels previously reported findings [20], where PACAP 6-38 was described as a competitive antagonist to CARTp *in vitro*. Lin and colleagues [20] reported that CARTp-induced p-ERK expression in cultured, differentiated PC12 cells was competitively reversed by a ten-fold higher dose of PACAP 6-38 versus CARTp. Here, we tested PACAP 6-38 effects in a dose-range relationship vs. agonist corresponding to that used by Lin et al. [20]. Similar to Lin et al., we found that the ten-fold higher dose of PACAP 6-38 completely blocked CARTp effects (Fig. 1C).

It could be argued that the feeding effect of CARTp in theory could be secondary to a stimulation of VIP or PACAP receptors rather than to a competitive primary action on a putative CARTp-receptor. However, there is some evidence for separable receptors, as PACAP and CARTp display different action profiles. In contrast to full-length PACAP (summarized by Vaudry et al. [28]), CARTp does not affect gastrointestinal motility or acid secretion after peripheral administration [4], [5]. This supports the notion that CARTp is unlikely to be acting directly on PACAP receptors but rather on a CARTp-sensitive receptor structure.

In addition to exhibiting a primary effect to inhibit food intake [12], CARTp was shown in one previous study [16] to induce a conditioned taste aversion (CTA). In order to avoid the occurrence of a CTA, we used the maintenance chow diet as the testing stimulus and the drugs were given in random order using a change-over design to balance out any confounding effects. To further avoid the risk of carry-over effects after each testing day, there was a two-day wash-out period before the next treatment was given. The CARTp-induced food intake inhibition was seen to occur between 2 and 5 h after injection (Fig. 1), whereas the food intake rates were similar from 5 to 22 h after CARTp administration such that no compensatory increase occurred. Additionally, the CARTp-induced short-lasting changes in body weight (Fig. 2A) were followed by normalized weight gain (Fig. 2B). Together this indicates that the animals did not develop avoidance behavior to the diet. We cannot exclude the possibility that the reduction in food intake by CARTp presently observed was compounded by feelings of nausea or illness. However, PACAP 6-38 abolished CARTp-induced changes in food consumption regardless of the mechanisms contributing to the CARTp feeding effects.

Consistent with a previous report [29], our control experiments show that PACAP 6-38 had no significant effect on food consumption by itself under the present testing paradigm, either when given fourth i.c.v. (Table 1) or in the periphery (Table 2) in a dose-range effective to block CARTp effects (Fig. 1). PACAP 6-38 failed to produce any effect after s.c. administration, even after we used a dose two times greater (6 nmol) than the highest effective i.c.v. PACAP 6-38 dose. This further confirms that PACAP 6-38 blocks the CARTp effect at a central nervous level rather than causing peripheral confounding effects counteracting that elicited by centrally delivered CARTp.

In the absence of an antagonist to CARTp, Kristensen et al. showed that central administration of a CART peptide antiserum led to an increased food intake [12]. Based on this observation, and of differences in the levels of CART mRNA expression in feeding centers in fed vs. starved rats, endogenous CARTp was proposed to play a role in central nervous food intake regulation [12], [17], [30]. Access to a competitive CARTp antagonist will allow for Kristensen’s hypothesis to be tested pharmacologically. It should be emphasized that the present study was designed to establish if PACAP 6-38 interacts antagonistically *in vivo* with exogenous CARTp, and not to determine the possible role of endogenous CARTp in feeding. Here, the PACAP 6-38 was subsequently given to animals at the onset of the dark period when the drive to ingest is high, in order to detect an abolishment of exogenously delivered CARTp-induced feeding inhibition. Under this paradigm, centrally delivered PACAP 6-38 did not by itself affect food intake (Table 2). This does not exclude that PACAP 6-38 administered centrally by itself in fully or nearly satiated animals, in which the endogenous CART activity is high [12], [31], may well increase food intake as a consequence of possible CARTp receptor antagonism. Therefore, the lack of a PACAP 6-38 effect by itself under the current paradigm does not contradict the hypothesis of a role for endogenous CARTp in food intake regulation, but merely supports the suggestion that PACAP 6-38 acts antagonistically to CARTp at a central nervous level.

In experiment 1, fourth i.c.v. CARTp induced the motor manifestations typically described by others: ataxic walk, mild tremors, and a flat posture [2], [12], [14], [15], [27]. This typical behavior was present in animals receiving CARTp, as well as CARTp against all the doses of PACAP 6-38 in experiment 1, but not after PACAP 6-38 by itself (experiment 2). Some of the gross motor effects have been suggested to originate from direct or indirect CARTp-stimulation of central dopamine receptors [27] or to be due to a serotonergic syndrome [16]. Intraventricular delivery of CARTp induces a variety of behavioral and physiological phenomena, including inhibition of food intake, gastric emptying, and changes in motor behavior. Results from studies on specific targets of action, where directed local intraparenchymal infusions have been administered, indicate anatomically and functionally separable CARTp-receptor substrates. For example, Kimmel et al. [27] showed that CARTp affects locomotor behavior after intra-ventral tegmental area injection, but not after injection into the substantia nigra. Whereas Zheng et al. found no effect on feeding after dorsal vagal complex (DVC) CARTp delivery [19], we showed that infusions of CARTp into the DVC did inhibit gastric emptying [32]. In this latter study, no apparent changes in motor behavior were seen in response to CARTp in the DVC, although this was considered outside the scope of the investigation and was therefore not reported. The present findings suggest that the effects of CARTp are not only functionally but pharmacologically separable as well. The observation that PACAP 6-38 appears to block only some effects of intraventricular CARTp (food intake inhibition and short-term weight change but not changes in motor behavior), supports the hypothesis that there may be at least two different subtypes of CARTp-sensitive receptors, some of which appear less responsive to PACAP 6-38.

### Summary

Our present results show that PACAP 6-38 can act as a functional *in vivo* antagonist to CARTp 55-102 and block its effects on food intake and short-term weight change. PACAP 6-38 did not impact CARTp-induced changes in motor behavior suggesting that CARTp exerts its separable effects via multiple receptor subtypes. Although CARTp acts to inhibit food intake as shown in functional studies [2], [5], the precise anatomical target site(-s) for putative CARTp receptors involved in food intake inhibition have yet to be described. Thus, access to a CARTp antagonist effective *in vivo* is of great significance as new ligand candidates may help to firmly establish neuroanatomical location of putative CARTp receptors, and to further benefit investigations of endogenous CARTp action.
